# Neuropathic and nociceptive pain in head and neck cancer patients receiving radiation therapy

**DOI:** 10.1186/1758-3284-1-26

**Published:** 2009-07-14

**Authors:** Joel B Epstein, Diana J Wilkie, Dena J Fischer, Young-Ok Kim, Dana Villines

**Affiliations:** 1 University of Illinois Cancer Center, University of Illinois, Chicago, USA; 2 University of Illinois College of Nursing, University of Illinois, Chicago, USA; 3 University of Illinois College of Dentistry, University of Illinois, Chicago, USA; 4 Univerity of Illinois College of Medicine, University of Illinois, Chicago, USA

## Abstract

**Background:**

Pain is common in head and neck cancer (HNC) patients and may be attributed to the malignancy and/or cancer treatment. Pain mechanisms and patient report of pain in HNC are expected to include both nociceptive and neuropathic components. The purpose of this study was to assess the trajectory of orofacial and other pain during and following treatment, using patient reports of neuropathic pain and nociceptive pain and pain impact.

**Methods:**

124 consecutive HNC patients receiving radiation therapy (RT) (95 men, 29 women; mean age: 54.7 ± 12.3 years) participated in a patient-reported outcome (PRO) assessment. Patients completed the McGill Pain Questionnaire three times during therapy and 3 months following study entry.

**Results:**

The majority of patients related their pain to the tumor and/or cancer treatment. Whereas 59% reported their pain to be less severe than they expected, 29% were not satisfied with their level of pain despite pain management during cancer therapy. Worst pain was 3.0 ± 1.3 on a 0- to 5-point verbal descriptor scale. Pain intensity was present at entry, highest at 2-week follow-up, declining towards the end of treatment and persisting at 3-month follow-up. The most common neuropathic pain descriptors chosen were aching (20%) and burning (27%); nociceptive words chosen were dull (22%), sore (32%), tender (35%), and throbbing (23%), and affective/evaluative descriptors were tiring (25%) and annoying (41%). 57% of patients reported continuous pain, and combined continuous and intermittent pain was reported by 79% of patients.

**Discussion:**

This study provides evidence that patients with HNC experience nociceptive and neuropathic pain during RT despite ongoing pain management. The affective and evaluative descriptors chosen for head and neck pain indicate considerable impact on quality of life even with low to moderate levels of pain intensity. These findings suggest that clinicians should consider contemporary management for both nociceptive and neuropathic pain in head and neck cancer patients.

## Background

Pain is common for the 35,310 people who are diagnosed with head and neck cancer (HNC) in the United States annually [1,2]. HNC pain may arise due to tissue damage from multiple sources such as mucosal injury, invasion of the tumor into somatic tissue (skin, muscle, bone) with inflammation or ischemia, and nerve infiltration or compression [3]. Treatment for HNC involves single or multimodal therapy employing surgery, chemotherapy (CT) and/or radiation (RT), all of which can damage somatic tissues and nerves. These multiple sources of somatic tissue and neural damage from the tumor and cancer treatment result in pain being experienced by all HNC patients [4].

Neuropathic pain is defined by The International Association for the Study of Pain (IASP) as pain initiated or caused by a primary lesion or dysfunction in the nervous system resulting in debilitating pain [5]. Damage to somatic and primary and/or central neurons commonly associated with nociceptive pain may result in neuropathic pain [6-8]. This dysfunction in the nervous system may be exacerbated by persistent unrelieved nociceptive pain associated with the tumor or cancer treatments (e.g., mucositis), thereby producing neuropathic pain. Alteration in pain processing at peripheral sites (such as with mucositis) and central levels (that may occur when mucositis pain is persistent) produces characteristic sensory abnormalities such as hyperalgesia and allodynia [9]. Hyperalgesia is defined as an increased response to a stimulus that is normally painful and allodynia as pain due to a stimulus that does not normally provoke pain [8].

One of the most feared consequences of cancer is the possibility of severe and uncontrolled pain in patients with advanced cancer [10]. In patients with HNC, pain is reported in up to 85% of cases at diagnosis [11,12]. Pain due to soft tissue and bone destruction and nerve injury may involve inflammatory and/or neuropathic mechanisms [13-19]. Further, it is estimated that 45% to 80% of all cancer patients have inadequate pain management [20-22]. Barriers to adequate pain management include patients' reluctance to report pain [23], current pain management practices by health care providers [24] and providers' negative ideas about and regulatory barriers to the use of opioids [25]. In addition, limited understanding of the frequency and role of neuropathic pain mechanisms and the lack of use of management approaches for neuropathic pain may compromise symptom management in cancer patients.

Cancer pain causes increased morbidity, reduced performance status, increased anxiety and depression, and diminished quality of life (QOL) [17,26-28]. Head and neck and oral pain management may be particularly challenging due to the rich innervation of the orofacial region and because oral intake, swallowing, speech and other motor functions of the head and neck and oropharynx are constant pain triggers. In addition, the oral mucosa is susceptible to the effects of systemic chemotherapy and regional radiotherapy, resulting in painful mucositis. The oral microbial flora may cause secondary infection with attendant pain and morbidity.

Pain may be the first symptom in 20% to 50% of all cancer patients [10] due to the malignancy, and oral pain may arise from HNC in up to 85% [10,11] from metastatic disease in the head and neck or due to oral involvement in systemic cancers (e.g., leukemia). In a recent study, investigators identified pain in 56% of patients with HNC at diagnosis, and found mixed nociceptive and neuropathic pain in 93% of those with pain [29]. In a retrospective study of 1,412 patients with oral cancer, pain was identified as the first sign of cancer in 19%, and pain was commonly reported with tumor recurrence [30]. Others reported cancer-related pain in 52% of hospitalized patients, with pain directly due to tumor in 29% and to surgery in 50% [31,32]. In large surveys of pain characteristics in cancer including HNC [17,19], patients suffered pain associated with the tumor (87% to 92.5%), or cancer therapy (17% to 20.8%) or both. In HNC, 78% of patients report pain in the head, face or mouth and 54% in the cervical region or shoulder [19]. In HNC, pain is the major reason (up to 85%) for seeking care [33], but at diagnosis pain is usually of low intensity (mean 10-cm visual analogue scale [VAS] = 3). Orofacial pain associated with cancer management is a well-recognized adverse effect of treatment, but whether this represents nociceptive or neuropathic mechanisms is not well defined. Pain due to oral mucositis is the most frequently reported patient-related complaint impacting QOL during cancer therapy [34-42] and often results in severe pain for which opioid analgesics are prescribed [38,43-45], sometimes with additional impaired QOL. Successful pain management requires knowledge of, and attention to, multiple pain mechanisms that may culminate in the patient's pain.

In HNC patients, neuropathic pain has not been well characterized in terms of sensory report (location, intensity, quality and pattern) or sensory quantification (allodynia and hyperalgesia). Grond et al. [46] reported that 30% of HNC patients suffered from neuropathic pain as result of the cancer or its treatments. In addition to neuroplasticity as a mechanism for neuropathic pain, other mechanisms may play a role in producing neuropathic pain associated with mucositis that may be conditioned by inflammatory mediators (e.g., tumor necrosis factor-alpha [TNF-α]), which play a central role in the activation of cytokines and are elevated in mucositis [47]. TNF-α is known to be involved in mediation of neuropathic pain and hyperalgesia [48]. Other chemical mediators implicated in neuropathic pain include reactive oxygen/nitrogen species, bradykinin, substance P and other cytokines that are upregulated in mucositis [47]. Investigators also have demonstrated changes in dorsal horn processing of nociceptive stimuli that result in neuropathic pain [49]. These mechanisms may result in neuropathic pain associated with tissue damage that occurs from HNC or its treatment. Previous investigators have not characterized the pain in HNC patients using multidimensional pain measures. The purpose of this paper is to describe the experience and trajectory of sensory pain (location, intensity, quality, and pattern) in patients with HNC undergoing cancer treatment using PRO, including neuropathic and nociceptive pain descriptors.

## Methods

We conducted a 3-month repeated-measures study to describe the trajectory of pain and pain descriptors in consecutive patients with HNC. The study was approved by the Institutional Review Board at the University of Washington for initial data collection and at the University of Illinois at Chicago for ongoing data analysis.

### Sample

Eligible subjects: (a) had a diagnosis of HNC; (b) spoke and read English; and (c) had pain related to the cancer or to cancer therapy during the week prior to enrolling. Patients were excluded if they: (a) had surgery within one month; (b) were physically unable to complete study procedures; or (c) were mentally unable to complete study questionnaires because of brain metastases or developmental problems, as measured by a Mini-Mental State Exam (MMSE) [50] (defined as a score of 20 or less). The MMSE is an 11-question scale designed to efficiently screen a person's cognitive functioning. Scores 19 and below represent cognitive impairment [50].

Of the 151 patients eligible for the study, 27 refused and 124 consecutive patients participated (Table 1). The participants included 95 men and 29 women. Their mean age was 54.7 ± 12.3 years. Most of the participants were Caucasian (88%), with 3% Hispanic and 2% African American. Other demographics of the patients are shown in Table 1. The primary tumor site was oral cavity/oropharynx (46%), major salivary gland (23%), maxillary sinus (12%), larynx (11%), and unknown (8%). The tumor stage at enrollment was Stage I in 17%, Stage II in 14%, Stage III in 14% and Stage IV in 46%; 10% were unknown primary. The histologic diagnoses were: adenocarcinoma (13%); adenoid cystic carcinoma (31%); mucoepidermoid carcinoma (13%); squamous cell carcinoma (37%); and miscellaneous (7%). Most (68.5%) patients had surgery prior to radiation therapy.

**Table 1:** Demographic characteristics of enrolled subjects (N = 124)

**Variable**	**Category**	**Frequency (n)**	**Percent**
Gender	Male	95	77%
	Female	29	23%

Education	<= 8^th ^grade	3	2%
	12^th ^grade	54	44%
	Associated degree	32	26%
	>= Bachelor's degree	30	24%
	Missing	5	4%

Ethnicity	Caucasian	109	88%
	African American	3	2%
	Hispanic	4	3%
	Asian	3	2%
	Other	5	4%

Cancer Stage (Current)	Stage I	22	18%
	Stage II	17	14%
	Stage III	15	12%
	Stage IV	62	50%
	Missing	8	6.5%

Surgery	No	38	31%
	Yes	85	69%
	Missing	1	1%

Chemotherapy	No	91	73%
	Yes	31	25%
	Missing	2	2%

Radiation Therapy	Yes	124	100%

Origin of Pain	Tumor-related	26	21%
	Treatment-related	66	53%
	Both	24	19%
	Unknown	8	6%

### Procedures

Medical and dental providers in the cancer clinic introduced patients to the investigators in person. A research team member informed patients about the study and scheduled data collection to coincide with a scheduled clinic appointment if patients were eligible and agreed to participate. The researcher obtained a signed informed consent, administered the MMSE to confirm eligibility, and interviewed the patient to complete a demographic data form. Patients were seen by their oncology providers for routine clinic follow-up visits. After the clinic visit, patients completed the valid and reliable 1970 version of the McGill Pain Questionnaire [51] at 2 weeks, 4 weeks and 3 months after the baseline measures to record pain in the extra-oral and intra-oral environments. Pain location was measured as the number of pain sites marked on a body outline. Pain intensity was measured as current, least, and worst pain using the 0–5 verbal descriptor scale. Pain quality was measured as descriptors selected from a list of 78 that represented sensory (PRI-S), affective (PRI-A), evaluative (PRI-E), miscellaneous (PRI-M), and total (PRI-T) pain, as well as number of words chosen (NWC) [52-54]. Pain pattern was measured as descriptors selected from a list of nine words representing continuous, intermittent, and brief patterns of pain. The following information was recorded from interview or medical record review: (1) gender; (2) age; (3) diagnosis(es); (4) medication(s) taken, including systemic and topical anesthetics and analgesics and time of last dose; and (5) RT dose and CT agent(s).

Research staff members entered data in CRUNCH4 (Crunch Software Corporation, San Francisco, CA) and exported it to SPSS (SPSS, Inc., Chicago, IL) for data analysis. We present descriptive statistics for the pain location, intensity, quality, and pattern, as well as the number of nociceptive and neuropathic descriptors reported at each measurement time point. Pain quality findings are reported for the first pain site reported that was located in the head and neck region. We plotted scores and calculated repeated-measures ANOVAs over time for pain scores reported during the 3-month study.

## Results

Patient characteristics are shown in Table 1. Cancer therapy delivered was radiation therapy alone (n = 21; 17%), a combination of surgery and radiation therapy (n = 70; 57%), a combination of chemotherapy and radiation therapy (n = 17; 14%), or a combination of surgery, radiation and chemotherapy (n = 14; 11%).

Prior to study entry, the majority of patients reported pain for 0–6 months (77%), 7–12 months (5.6%), 13–23 months (4%), or more than 2 years (12.9%). At baseline, patients associated their pain with their cancer (21%), surgery (53.2%), or both tumor and surgery (20.5%). On average, patients reported 2.1 ± 2.2 pain sites. Pain measures at baseline are shown in Table 2. The distributions of the pain locations are presented for the first site marked and for all sites marked (Table 3). The neuropathic, nociceptive, affective, and evaluative pain descriptors are shown in Table 4.

**Table 2:** Pain measures at baseline

**Variable**	**Category**	**Frequency (n)**	**%**	**Mean**	**SD**	**Min/Max**
Pain expectation	Worse than expected	18	15			
	The same as expected	28	23			
	Not as bad as expected	65	59			

Satisfied with Pain Level	No	36	29			
	Yes	80	65			
	Missing	8	7			

Current pain (0–5)				1.51	1.01	0–5

Worst pain (0–5)				3.03	1.26	0–5

Least pain (0–5)				0.76	0.74	0–3

Number of pain sites				2.1	2.2	0–16

Pain Rating Index	Sensory			9.1	7.3	0–34
	Affective			1.0	1.7	0–8
	Evaluative			1.2	1.5	0–5
	Miscellaneous			2.1	2.9	0–15
	Total			13.5	11.3	0–58

Number of Words Chosen				5.9	4.1	0–20

Pain Pattern	Continuous	41	33.1%			
	Intermittent	18	14.5%			
	Brief	2	1.6%			
	Continuous/Intermittent	16	12.9%			
	Continuous/Brief	2	1.6%			
	Intermittent/Brief	26	21%			
	Continuous/Intermittent/Brief	11	8.9%			
	Missing	8	6.4%			

**Table 3:** Frequency of pain location areas as marked on a body outline for the first site marked and for all pain sites (ranged from 1 to 6 pain sites for each of the 124 participants)

**Pain Location Area**	**Frequency for first site marked**	**Frequency for all 6 pain sites marked**
Head (head, neck, face, chin, gum/tongue, chin, mouth)	98	101

Arm (arm, shoulder, elbow, hand)	3	12

Chest (chest, breast)	0	3

Abdomen	1	11

Leg (leg, knee, foot)	0	3

Back (back, spine)	3	6

Buttock (buttock, hip, anus)	0	6

Missing/unknown	19	0

**Table 4:** Frequency of pain quality descriptors attributed to head and neck pain site

**Neuropathic Descriptor**	**Frequency (%)**	**Nociceptive Descriptor**	**Frequency (%)**	**Affective and Evaluative Descriptors**	**Frequency (%)**
Aching	**25 (20.2%)**	Beating	4 (3.2%)	Fearful	5 (4.0%)

Boring	7 (5.6%)	Cramping	0 (0%)	Frightening	3 (2.4%)

Burning	**33 (26.6%)**	Crushing	3 (2.4%)	Terrifying	0 (0%)

Cold	0 (0%)	Cutting	4 (3.2%)	Grueling	4 (3.2%)

Cool	1 (0.8%)	Dull	**27 (21.8%)**	Punishing	3 (2.4%)

Drawing	1 (0.8%)	Gnawing	6 (4.8%)	Cruel	0 (0%)

Drilling	3 (2.4%)	Heavy	2 (1.6%)	Vicious	2 (1.6%)

Flashing	5 (4.0%)	Hurting	16 (12.9%)	Killing	1 (0.8%)

Flickering	10 (8.1%)	Lacerating	5 (4.0%)	Tiring	**31 (25%)**

Freezing	1 (0.8%)	Piercing	11 (8.9%)	Exhausting	10 (8.9%)

Hot	9 (7.3%)	Pinching	2 (1.6%)	Wretched	2 (1.6%)

Itchy	0 (0%)	Pounding	2 (1.6%)	Blinding	1 (0.8%)

Jumping	4 (3.2%)	Pressing	13 (10.5%)	Sickening	0 (0%)

Lancinating	1 (0.8%)	Pulling	0 (0%)	Suffocating	2 (1.6%)

Numb	14 (3.2%)	Pulsing	9 (7.3%)	Annoying	**51 (41.1%)**

Penetrating	1 (0.8%)	Rasping	10 (8.1%)		

Pricking	2 (1.6%)	Sharp	19 (15.3%)		

Quivering	4 (3.2%)	Sore	**40 (32.3%)**		

Radiating	1 (0.8%)	Splitting	4 (3.2%)		

Scalding	2 (1.6%)	Squeezing	3 (2.4%)		

Searing	4 (3.2%)	Taut	8 (6.5%)		

Shooting	9 (7.3%)	Tearing	6 (4.8%)		

Smarting	7 (5.6%)	Tender	**43 (34.7%)**		

Spreading	2 (1.6%)	Throbbing	**29 (23.4%)**		

Stabbing	11 (8.9%)	Tugging	3 (2.4%)		

Stinging	0 (0%)	Wrenching	0 (0%)		

Tight	0 (0%)				

Tingling	12 (9.7%)				

	Min = 0		Min = 0		Min = 0
	Max = 11		Max = 15		Max = 13
	Mean = 2.5		Mean = 2.9		Mean = 2.2
	SD = 2.3		SD = 2.5		SD = 2.4

**Figure 1:**
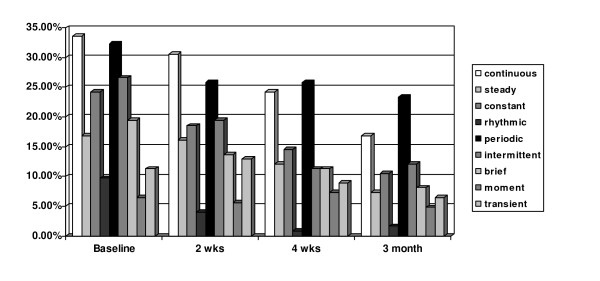
**Frequencies of Each Pain Pattern Descriptor (%)**.

The pain intensity scores presented in Table 2 indicate that the average pain intensity was mild and continued despite pain management interventions, yet 29% of the patients were not satisfied with their level of pain. Head and neck and oral were the most common sites of pain during RT (79% of patients). On average, patients selected 1.5 ± 1.8 neuropathic words (min = 0, max = 9), with burning selected as the most common descriptor (21%). They selected 1.8 ± 2.1 nociceptive words (min = 0, max = 14), and the most common were tender (26.6%), soreness (25%), and throbbing (20.2%). Affective pain quality descriptors selected were: tiring (22.6%), nagging (17.7%), nauseating (7.3%) and exhausting (7.3%). Evaluative descriptors were: annoying (35.5%), troublesome (8.9%), and miserable (8.9%). The average PRI-T was 13.5 ± 11.3. Patients reported their pain pattern was constant (65.5%), intermittent (57.3%), and/or transient (33.1%) (Figure 1).

**Figure 2:**
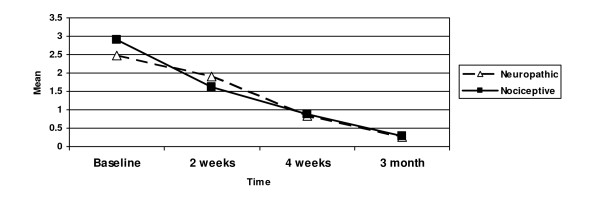
**Mean Number of Neuropathic and Nociceptive Words**.

**Figure 3:**
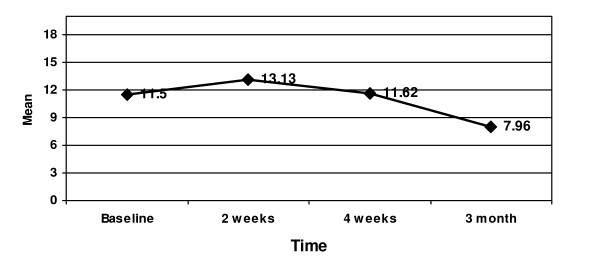
**Mean Pain Rating Index: Total at Baseline, 2 weeks, 4 weeks, and 3 months**.

Pain scores decreased from admission to last follow-up. ANOVA showed significant decreases in the pain measures over the 4 time points: the number of the neuropathic words chosen (*F*(_3,222_) = 48.5, *p *< 0.001); the number of the nociceptive words chosen (*F*(_3,222_) = 51.2, *p *< 0.001) (Figure 2); PRI-S (*F*(_3,222_) = 11.9, *p *< 0.001); PRI-A (*F*(_3,222_) = 1.5, *p *< 0.21); PRI-E (*F*(_3,222_) = 3.5, *p *< 0.02); PRI-M (*F*(_3,222_) = 2.6, *p *< 0.05); PRI-T (*F*(_3,222_) = 9.2, *p *< 0.001) (Figure 3); and total NWC (*F*(_3,222_) = 8.7, *p *< 0.001).

**Figure 4:**
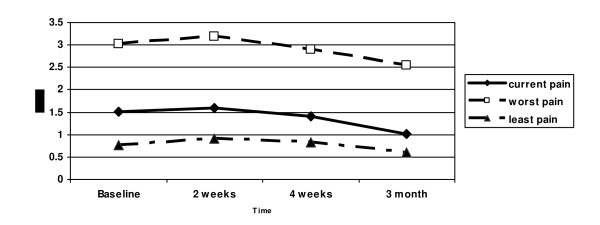
**Mean Scores for Current, Least, and Worst Pain Intensity at Baseline, 2 weeks, 4 weeks, and 3 months (0–5 scale)**.

Current pain at visit one was 1.51 ± 1.01, worst pain was 3.03 ± 1.26; those increased at time 2, where current pain was 1.60 ± 0.81 and worst was 3.18 ± 1.15, and decreased at subsequent time points. The scores for current and worst pain, pain pattern, and number of pain sites were not significantly different by the type of cancer therapies that the patient received. The subjects with both chemotherapy and radiation treatments reported statistically significant higher PRI-T scores (mean, 19.58 vs. 12.56, t = -2.29, *p *< .024 than the subjects with other therapies (e.g., radiation only, surgery and radiation; or chemotherapy, radiation and surgery). At time 3, worst pain was 2.89 ± 1.23, and at time 4 worst pain was 2.54 ± 1.40, current pain 1.02 ± 1.07 and least pain 0.60 ± 0.81 (Figure 4). A highly correlated linear trend was seen between the number of nociceptive and neuropathic words chosen during treatment. Patient demographics, including age, gender, ethnicity and income, did not correlate with the variables assessed.

These reports of pain continued despite use of analgesics and adjuvant drugs for pain management during and following cancer therapy. Of these patients, 10.5% were taking step 1 analgesics (NSAIDs and non-opioids), 21% adjuvant analgesics, 8.1% step 2 opioids (for mild to moderate pain intensity), 20.2% step 3 opioids (for moderate to severe pain intensity). The other patients were taking combinations of analgesics and adjuvant drugs: 8.9% adjuvant and step 1 analgesics; 3.2% adjuvant and step 2 opioids; 4% adjuvant and step 3 opioids; 6.5% step 1 and step 2 opioids; 1.6% step 2 and step 3 opioids; 1.6% adjuvant, step 1, and step 2 opioids; 0.8% adjuvant, step 1 and step 3 opioids; and 11% were missing analgesic drug data.

We estimated adequacy of analgesic prescription by calculating a pain management index (PMI) as suggested by Cleeland [55,56]. We subtracted the worst pain level score from the analgesics step score to produce a PMI score. A negative PMI score indicated inadequate analgesic prescription (under-treatment of pain) and 0 or positive PMI scores indicated satisfactory analgesics. Based on Cleeland's PMI, 63.7% of the patients were taking prescriptions satisfactory for their pain intensity level; 23.4% were taking prescriptions that represented undertreatment for the level of their pain intensity. PMI scores could not be calculated for those for whom drug data were missing.

## Discussion

HNC patients were enrolled in the study during cancer treatment when pain was reported. Patients attributed their pain to the cancer, prior surgical treatment or the ongoing radiation therapy for HNC. Pain was discomforting on average at entry (worst pain intensity 3.0 ± 1.3), and it was less than anticipated in more than half of the patients, as expected in about one-fifth and worse than expected by some. In our survey, pain was reported first in the head and neck or oral cavity by 79% (n = 98) of the participants.

In a previous study, pain was reported by patients with oral squamous cell carcinoma at presentation in 39% of 138 cases and correlated with tumor stage [57]. Pain at diagnosis of HNC has been variable, with pain reported in up to 85% of patients [58]. Investigators of one study found that 65.5% of 1,070 cancer patients reported pain prior to cancer therapy, whereas pain was less common at diagnosis in another study (48.1%) [59,60]. In a recent study, pain was identified in 56% of patients with HNC at diagnosis [29]. Pain at diagnosis is typically of low intensity discomfort as the first symptom leading to diagnosis [58]. The most common qualitative descriptions of pain were aching, dull, or pressing [33]. Interestingly, patients who present with pain before treatment develop significantly higher impairment scores due to pain during and following treatment [59], suggesting that sensitization occurs. We found similar findings in our trial, where report of nociceptive and neuropathic pain at entry predicted pain experience during and following therapy.

Neuropathies are commonly reported in patients with malignancy (1.7%–5.5%) and may be due to direct effects of the tumor, paraneoplastic syndromes and/or treatment-related toxicity [61-64]. Neurotoxicity is increased in patients with pre-existing nerve damage [65] and with nutritional deficiencies [66]. However, the incidence of paraneoplastic neuropathies occurring in the orofacial region is unclear. In our survey, neuropathic descriptors of pain were selected by a total of 73% of subjects (n = 91), suggesting that neuropathic pain is common in patients with HNC. In a previous study, mixed nociceptive and neuropathic descriptors were chosen by 93% of HNC patients reporting pain at diagnosis [29]. The affective and evaluative impact of pain in head and neck and oral sites in these patients indicates the significant impact of head and neck pain, in which neuropathic mechanisms are common. The finding of a linear trend in the number of nociceptive and neuropathic words chosen during treatment suggests that the pain experience may be due to both nociceptive and neuropathic pain. Patient demographics, including age, gender, ethnicity and income, did not correlate with the variables assessed. Approximately one-third of patients reported continuous pain, 40% reported continuous plus intermittent pain and 15% reported intermittent pain associated with oral function such as with eating or swallowing. We did not identify a shift from neuropathic and nociceptive word choice during treatment, suggesting that both mechanisms are associated with pain at entry and during RT.

The most common acute oral side effect of chemotherapy and radiotherapy is oral mucositis [67]. Oral mucositis and associated pain is reported to be the most distressing symptom in radiotherapy, with increasing pain intensity and pain interference scores by week 3, peaking at week 5 [68] and persisting for weeks following irradiation [35]. Mucositis pain is common (58%–75%) and interferes with daily activities in approximately one-third of subjects [69-74] and with social activities and mood in 50%–60% [35]. Combined chemotherapy and radiation therapy results in increased frequency, severity and duration of mucositis [73-76]. These findings were reflected in the pain report of subjects in this study, where pain intensity remained essentially unchanged during RT despite use of analgesics and other pain management. In addition to mucositis, some cytotoxic agents may cause jaw pain and neuropathy (e.g., vincristine, vinblastine, platinum).

Surgical procedures commonly result in acute nociceptive orofacial pain and establish conditions that may lead to painful post-surgical neuropathy. In addition to tissue injury at tumor resection, morbidity is increased by concomitant procedures such as radical neck dissection [77]. In this study, patients reported that pain was related to the tumor (21%), and related to cancer treatment (53.2%) or both (20.5%), indicating that patients felt that the majority of pain was treatment-related. Resection of the mandible inevitably leads to sensory impairment [78], with 50% of recipients experiencing regional hyperalgesia or allodynia. At 2–5 years post maxillectomy, 88%–90% of patients reported persistent pain [79]. In an analysis of patients treated for laryngeal cancer, ablative surgery with adjuvant chemo- and/or radiotherapy was associated with increased chronic pain and psychosocial morbidity compared to that of patients treated by chemoradiation alone [80], underscoring the impact of surgical intervention. Investigators of another study, examining pain scores following HNC surgery, showed that the highest scores were for the oral cavity, followed by the larynx, oropharynx and nasopharynx [79]. In a large survey of surgically treated oral cancer patients, functional problems were reported postoperatively in more than 50% of cases [60]. At review (= 6 months post-surgery), impairment due to moderate to severe pain was found in 34.3% of cases [60]. In two studies, the most frequent pain locations were the shoulder (31%–38.5%), neck (4.9%–34.9%), TMJ (4.9%–20.1%), oral cavity (4.2%–18.7%) and the face and other head regions (4.2%–15.6%) [60,81], reflecting morbidity secondary to surgical management [77,82]. Fortunately, there is a tendency for post-treatment symptoms to improve with time [83]. By 60 months post surgery, a smaller proportion of patients (14.9%) (n = 74) had persisting pain [81]. In cancer patients, the postoperative pain experience typically is characterized by acute pain persisting 1–2 months, with a gradual improvement over time [83-85]. However, long-term HNC survivors (> 3 years) still suffer significantly more pain and functional problems than matched control subjects, even though there is a relative return towards normal function [81,83,85]. Persisting pain following surgery may involve inflammatory and neuropathic pain mechanisms, depending on the extent of surgery and its anatomic location. Functional consequences are often secondary to pain and may involve wound contractures and scarring [81]. Our study provides evidence that patients with HNC experience nociceptive and neuropathic pain. These findings are supported in prior studies, where 30% of HNC patients suffered from neuropathic pain in one study [46], and the majority of patients reported pain in another study [29].

The affective and evaluative descriptors chosen for head and neck pain indicate considerable impact on quality of life, even with low to moderate levels of pain intensity. Effective management requires accurate diagnosis of the multifaceted etiology of orofacial pain in cancer patients [47,86]. Pain intensity scores did not progress during treatment with ongoing pain management and were lower than at entry at the final assessment visit following RT. The findings suggest that expert medical management during cancer therapy can modulate the pain experience, despite the impact of radiation and chemotherapy. The PMI indicated that 63.7% of the patients were taking prescriptions satisfactory for their pain intensity level, while 23.4% were not adequately treated for their pain level; 10.5% of patients were on step 1 analgesics (non-opioid analgesics), 8.1% step 2 opioid (mild opioids), 20.2% step 3 opioids and 21% prescribed adjuvant analgesics. The other patients were using combinations of analgesics and adjuvant drugs.

Nociceptive pain is managed with treatment of the cause and topical anesthetics and analgesics, with reliance upon systemic analgesics. Neuropathic pain is typically more difficult to manage and in contrast relies upon locally acting anesthetics and centrally acting antidepressant and anti-convulsant medications, along with biopsychosocial treatment and systemic analgesics. Future research regarding pain in head and neck cancer patients should consider neuropathic and nociceptive pain-related complaints along with quantitative sensory testing to confirm neuropathic pain. In clinical practice, clinicians should consider contemporary management for both nociceptive and neuropathic pain in head and neck cancer patients.

## Conclusion

Pain experienced during radiation therapy for head and neck cancer is common. Neuropathic pain descriptors were chosen by 73% of patients and a linear trend was seen in number of neuropathic and nocicpetive descriptors chosen by pateints during therapy. Pain was common despite ongoing pain management during therapy. This study shows that pain during radiation therapy have both nocicpetive and neeuropathic qualities.

## Competing interests

The authors declare that they have no competing interests.

## Authors' contributions

JE reviewed study data and drafted the manuscript. YOK performed the statistical analysis. DJW conceived of the study, and participated in its design and coordination and helped to draft the manuscript. All authors contributed to and approved the final manuscript.
